# The relationship between home and community-based healthcare services utilization and depressive symptoms in older adults in rural China: a moderated mediation model

**DOI:** 10.1186/s12889-023-15590-2

**Published:** 2023-05-30

**Authors:** Zishuo Huang, Tingke Xu, Rujia Zhang, Xinxin Zhang, Shanshan Wang, Jiayun Zhang, Qingren Yang, Yating Fu, Jia Gui, Xiangyang Zhang, Chun Chen

**Affiliations:** 1 School of Innovation and Entrepreneurship, Wenzhou Medical University, Wenzhou, 325035 Zhejiang China; 2 School of Public Health and Management, Wenzhou Medical University, Wenzhou, 325035 Zhejiang China; 3 The 2 nd School of Medicine, Wenzhou Medical University, Wenzhou, 325035 Zhejiang China; 4 First Affiliated Hospital of Wenzhou Medical University, Wenzhou, 325035, 325000 Zhejiang China

**Keywords:** Home and community-based healthcare services utilization, Marital status, Instrumental activities of daily living, Depressive symptoms, Moderated mediation model

## Abstract

**Background:**

Studies have shown a close association between home and community-based healthcare services (HCBHS) utilization and depressive symptoms in older adults. However, no studies have explored the underlying mechanism of this relationship in rural China. This study was designed to evaluate the roles of instrumental activities of daily living (IADL) and marital status in the association between HCBHS utilization and depressive symptoms in Chinese rural older adults.

**Methods:**

Data were obtained from the 2018 China Health and Retirement Longitudinal Study, and 5,981 rural respondents (≥ 60 years old) were included. Depression scores were calculated using the ten-item Center for Epidemiological Studies Depression Scale. Moderated mediation analysis was carried out applying Hayes’ PROCESS macro (Model 7).

**Results:**

HCBHS utilization had a direct and negative effect on depressive symptoms. Furthermore, marital status moderated the association between HCBHS utilization and IADL, which belonged to the indirect influence of the first half on the association between HCBHS utilization and depressive symptoms. HCBHS utilization was associated with IADL in single but not in married respondents.

**Conclusion:**

The results demonstrated that marital status moderated the indirect relationship between HCBHS utilization and depressive symptoms, with HCBHS utilization being negatively associated with IADL among single but not married respondents. The government should focus on rural older adults, especially those who are single and have poor IADL function, and improve the provision of HCBHS to alleviate depressive symptoms.

**Supplementary Information:**

The online version contains supplementary material available at 10.1186/s12889-023-15590-2.

## Introduction

Depression, a common mental illness worldwide, is often characterized by low mood (unhappiness, agitation, or loneliness), poor concentration, a lack of interest in activities, and excessive guilt, which gives rise to undue burdens of disease [1]. Approximately 5.7% of adults aged 60 years or over suffered from depressive symptoms globally as of 2021 [1]. This may cause the affected older adults to behave in a dull state daily, such as being unable to eat, remaining in bed, or sitting on a stool all day without interacting with others [2, 3]. In the worst-case scenario, depressive symptoms can result in suicide. In China, around 36.14% of older adults were living with some extent of depressive symptoms in 2018 [4], which was more significant than in some European countries [5]. Moreover, Chinese rural older adults have an increased likelihood of suffering from depression relative to their urban counterparts [6–8]. Hence, concerns regarding mental health issues among older adults in rural China cannot be shrugged off lightly.

Some studies have shown that home and community-based healthcare services (HCBHS) play a vital role in ameliorating mental health status by reducing depressive symptoms and loneliness among community-dwelling older adults [9, 10]. Although previous studies suggested that HCBHS were closely linked to depressive symptoms in older adults worldwide, the mechanisms underlying this association remain unclear. It is of utmost significance to explore the moderated mediation pathways between HCBHS utilization and depressive symptoms in Chinese rural older adults to develop effective interventions for depressive symptoms.

Studies have shown that rural older adults in China had lower socioeconomic status, poorer self-rated health, and an increased risk of instrumental activities of daily living (IADL) disabilities compared with their urban counterparts, which led them to remain in stressful situation (e.g. low income and poor personal health status) [11–13]. IADL involving activities such as laundry, cooking, money management, housekeeping, and transportation, is more indicative of older adults’ ability to live independently and socially in the community compared to basic activities of daily living (BADL) [14–16]. Higher IADL scores always indicate a higher risk of late-life depression [17, 18]. Furthermore, recent research also found that access to HCBHS might have a significantly negative correlation with IADL disabilities among rural community-dwelling older adults in China [19]. However, the relationship between HCBHS utilization, IADL, and depressive symptoms has not yet been conclusively established in China’s rural areas.

In the face of growing rural hollowing-out, the majority of the young or middle-aged workforce migrates to urban areas for work [20], thus rural older adults mainly rely on the companionship and care of their spouses for social support [21]. Married rural older adults tend to receive more financial, physical, emotional, and instrumental assistance, contributing to lower IADL disabilities compared with those without a spouse [22, 23]. Moreover, married rural older adults tend to use less HCBHS, as they receive relatively more social support. Is it likely that the lower HCBHS utilization, the less relief there is for IADL disabilities? Therefore, is there a moderating effect of marital status on the relationship between HCBHS utilization and IADL scores, which is an indirect influence of the relationship between HCBHS utilization and depressive symptoms in rural older adults in China?

**Fig. 1:**
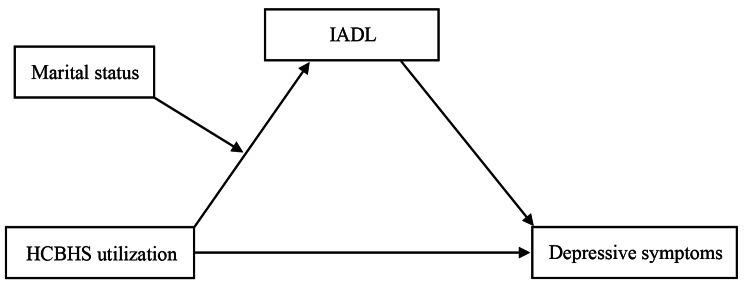
Hypothetical model Note: HCBHS, home and community-based healthcare services; IADL, instrumental activities of daily living

This study concentrated on community-dwelling older adults in China’s rural areas and applied a moderated mediation model to examine the association between HCBHS utilization and depressive symptoms. We hypothesized that marital status moderates the mediating effect of IADL on the association between HCBHS utilization and depressive symptoms in community-dwelling older adults in rural China. This hypothesis is illustrated in Fig. 1.

## Methods

### Data sources

The data used in the study were collected from the 2018 China Health and Retirement Longitudinal Study (CHARLS), a nationwide, ongoing, and large-scale social survey project designed by the National Development Research Institute of Peking University and the China Social Science Research Centre of Peking University. The national baseline survey used a stratified multi-stage sampling method, with counties and districts (both urban and rural) as the primary sampling units, to investigate 19,816 middle-aged and older adults aged 45 and above. The sample covered 450 villages and dwellings in 150 counties and districts in 28 provinces of mainland China, excluding Tibet. The response rate of respondents exceeded 80%, including 94% in rural areas and 69% in urban areas.

Respondents were excluded if they (a) were younger than 60 years old (**8,762**), (b) lived in areas other than rural areas (**2,967**), and did not match the data of (c) the ten-item Center for Epidemiological Studies Depression (CES-D-10) (**2,101**) and (d) IADL (**5**). A final sample of **5,981** rural community-dwelling older adults in China was included in this study.

#### Materials

##### Dependent variable: depressive symptoms

Designed to follow the contents of CES-D-10, The CHARLS questionnaire applied a 10-question depression scale to measure respondents’ psychological status [24], which was evaluated using a four-point Likert scale [25]. We scored the frequencies of depressive symptoms produced by the participants in the past week as 0 (rarely), 1 (some days: 1 or 2 days per week), 2 (occasionally: 3 or 4 days per week), or 3 (most of the time: 5 or 7 days per week), of which eight items were negative and two items were positive emotions. After reversing the scores of the positive feelings items and summarizing the total items, the scores of depressive symptoms ranging from 0 to 30 manifest that lower scores are associated with lower levels of depressive symptoms.

##### Independent variable: HCBHS utilization

HCBHS were measured in the CHARLS by asking respondents whether they used the following healthcare services provided by the community: (a) regular physical examinations, (b) on-site visits, (c) family beds, (d) community nursing, and (e) health management. We excluded “day care centers, nursing homes, senior dining tables, etc” and “entertainment” in the CHARLS as they should be included in daily care and social support services respectively [26–28]. This study aims to examine the differences in depressive symptoms in HCBHS users and non-users and explore the underlying mechanism of the relationship between HCBHS utilization and depressive symptoms. Hence, we classified respondents under “HCBHS utilization” if they utilized one or more services, coded 1; and under “no HCBHS utilization” otherwise, coded 0, listing 0 as the reference [29, 30].

##### Moderating variable: marital status

Respondents were asked: “What is your marital status?” The choices included: (a) married and cohabiting with a partner currently, (b) married but not cohabiting together due to work etc., (c) never married, (d) divorced, (e) widowed, and (f) separated. Respondents were stratified as “married” if they had a partner regardless of whether they were living together, coded 1; and as “single” otherwise, coded 0, listing 0 as the reference.

##### Mediating variable: IADL

The IADL reflects the basic competencies encompassing that older adults age and live in the community independently [31]. The IADL scale included five items in the CHARLS: cooking, grocery shopping, doing household chores, taking medicine, and managing finances, which were validated in previous studies [32, 33]. Respondents who did not have any difficulty in performing one scale of IADL were scored as 1, those who had difficulty but could still do it were scored as 2, those who had difficulty and needed help were scored as 3, and those who could not do it at all were scored as 4. The total IADL scores ranging from 5 to 20 manifest that with higher scores, there were more serious IADL disabilities.

##### Covariates

Based on extant literature, we selected confounding factors having an implication for depressive symptoms [34, 35]. Socioeconomic characteristics included gender, age, education level, social insurance, and wage and bonus income. Covariates of health status included self-rated health. Health behaviors included drinking, smoking, and exercising.

### Statistical analysis

Stratified by marital status, frequencies, percentages, mean, and standard deviation were used for the descriptive analyses. Pearson’s correlation was applied to examine the relationship between distinct key variables. Moderated mediation analysis was carried out applying Hayes’ PROCESS macro (Model 7) given ordinary least squares (OLS) regression-based path analysis [36]. This analysis was also based on bootstrapping (10,000 bootstrap samples) using 95% confidence intervals. Gender, age, educational level, income, social insurance, self-rated health, smoking, drinking, and exercising were considered covariates. A moderated mediation model examined the impact of marital status on the mediated correlation between HCBHS utilization and depressive symptoms among the respondents. HCBHS utilization was regarded as the independent variable, depressive symptoms as the dependent variable, IADL as the mediated variable, and marital status as the moderated variable. All data analyses were performed applying IBM SPSS Statistics for Windows, version 26.0 (IBM Corp.).

## Results

### Descriptive statistics

This study included 5,981 rural respondents aged 60 years or above in China, of whom 4,886 (81.7%) were married and 1,095 (18.3%) were single. Rural community-dwelling older adults who used HCBHS accounted for 18.8% of the population. The respondents’ mean scores for depressive symptoms and IADL were 9.357 ± 6.770 and 6.137 ± 2.446, respectively. More details on the essential attributes of the respondents are displayed in Table 1.

**Table 1:** Descriptive statistics of the manifest variables

Variables	Total		Married		Single	
	N (%)	M ± SD	N (%)	M ± SD	N (%)	M ± SD
**Gender**		0.524 ± 0.500		0.560 ± 0.497		0.368 ± 0.483
Male	3134 (52.4%)		2731 (55.9%)		403 (36.8%)	
Female	2847 (47.6%)		2155 (44.1%)		692 (63.2%)	
**Age**		0.133 ± 0.340		0.098 ± 0.297		0.292 ± 0.455
60–75 years old	5184 (86.7%)		4409 (90.2%)		775 (70.8%)	
≥ 76 years old	797 (13.3%)		477 (9.8%)		320 (29.2%)	
**Education level**		0.311 ± 0.463		0.285 ± 0.451		0.430 ± 0.495
Literate	4118 (68.9%)		3494 (71.5%)		624 (57.0%)	
Illiterate	1863 (31.1%)		1392 (28.5%)		471 (43.0%)	
**Marital status**		0.817 ± 0.387		--		--
Married	4886 (81.7%)		--		--	
Single	1095 (18.3%)		--		--	
**Income**		0.149 ± 0.356		0.164 ± 0.370		0.081 ± 0.273
Yes	889 (14.9%)		800 (16.4%)		89 (8.1%)	
No	5092 (85.1%)		4086 (83.6%)		1006 (91.9%)	
**Social insurance**		0.969 ± 0.174		0.975 ± 0.157		0.942 ± 0.235
Yes	5794 (96.9%)		4763 (97.5%)		1031 (94.2%)	
No	187 (3.1%)		123 (2.5%)		64 (5.8%)	
**Self-rated health**		0.684 ± 0.465		0.695 ± 0.460		0.634 ± 0.482
Good, very good, fair	4092 (68.4%)		3398 (69.5%)		694 (63.4%)	
Poor, very poor	1889 (31.6%)		1488 (30.5%)		401 (36.6%)	
**IADL**	5981 (100%)	6.137 ± 2.446		6.048 ± 2.349		6.533 ± 2.803
**Smoking**		0.300 ± 0.459		0.311 ± 0.463		0.254 ± 0.435
Yes	1797 (30.0%)		1519 (31.1%)		278 (25.4%)	
No	4184 (70.0%)		3367 (69.0%)		817 (74.6%)	
**Drinking**		0.331 ± 0.471		0.351 ± 0.477		0.244 ± 0.430
Yes	1980 (33.1%)		1713 (35.1%)		267 (24.4%)	
No	4001 (66.9%)		3173 (64.9%)		828 (75.6%)	
**Exercising**		0.891 ± 0.311		0.899 ± 0.301		0.858 ± 0.350
Yes	5332 (89.1%)		4393 (89.9%)		939 (85.8%)	
No	649 (10.9%)		493 (10.1%)		156 (14.2%)	
**HCBHS utilization**		0.188 ± 0.391		0.181 ± 0.385		0.217 ± 0.413
Yes	1123 (18.8%)		885 (18.1%)		238 (21.7%)	
No	4858 (81.2%)		4001 (81.9%)		857 (78.3%)	
**Depressive symptoms**	5981 (100%)	9.357 ± 6.770		8.993 ± 6.618		10.981 ± 7.191

Note. IADL, instrumental activities of daily living; HCBHS, home and community-based healthcare services; M, mean; SD, standard deviation

### Correlation analysis

The correlations among key research variables were calculated (Table 2). Married respondents showed lower likelihood of using HCBHS compared to single respondents (*r*=-0.036, *p* < 0.01). HCBHS utilization was not associated with IADL (*r*=-0.024, *p =* 0.059). HCBHS utilization was negatively linked to depressive symptoms (*r*=-0.034, *p* < 0.01). Married status was negatively related to IADL scores and depressive symptoms (*r*=-0.077, *p* < 0.001; *r*=-0.114, *p* < 0.001). Higher IADL scores were linked to more depressive symptoms (*r* = 0.314, *p* < 0.001). Details of all variables are displayed in Supplementary Table 1.

**Table 2:** Correlations among key variables (n = 5981)

Variables	1	2	3	4
1 HCBHS utilization	1			
2 Marital status	-0.036^**^	1		
3 IADL	-0.024	-0.077^***^	1	
4 Depressive symptoms	-0.034^**^	-0.114^***^	0.314^***^	1

Note: IADL, instrumental activities of daily living; HCBHS, home and community-based healthcare services; 1, HCBHS utilization; 2, Marital status; 3, IADL; 4, Depressive symptoms; *p < 0.05, **p < 0.01, ***p < 0.001

### Results of moderated mediation model

**Fig. 2:**
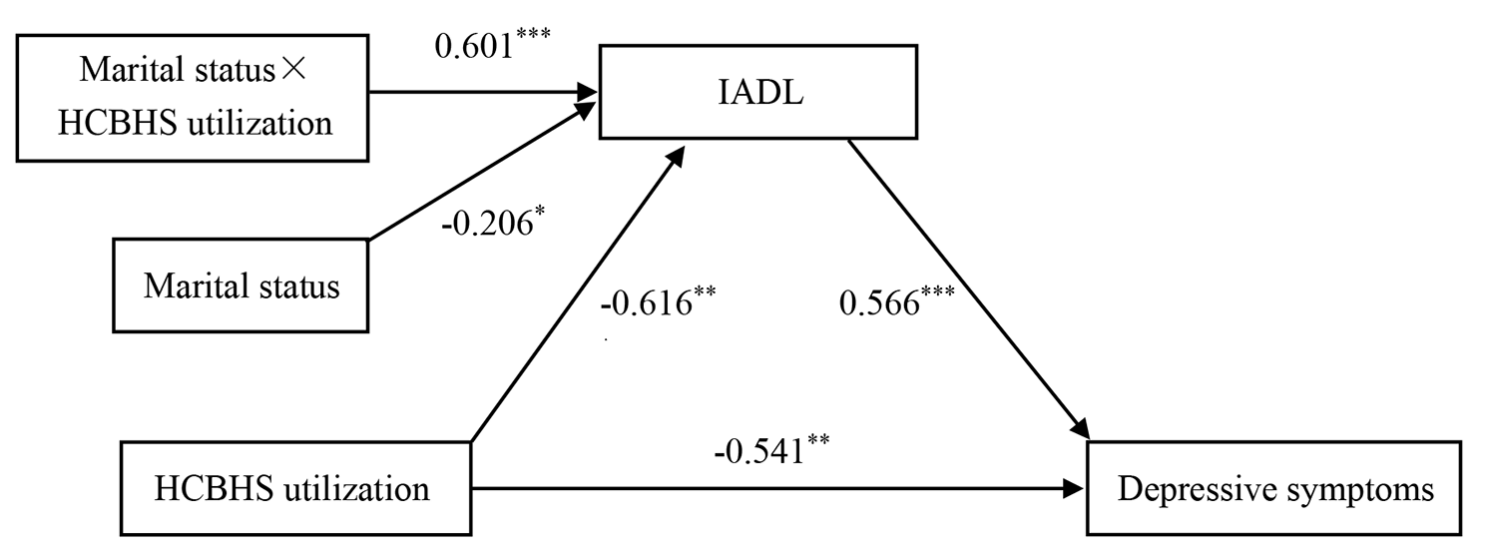
A moderated mediation model of the association between HCBHS utilization and depressive symptoms through IADL and marital status Note: Path coefficients are presented. HCBHS, home and community-based healthcare services; IADL, instrumental activities of daily living; ^*^p < 0.05, ^**^p < 0.01, ^***^p < 0.001

After adjusting for covariates, the pathway model of the moderated mediation analysis is shown in Fig. 2, corresponding to Table 3. The analysis indicated that HCBHS utilization (*B*=-0.616, *t*=-3.801, *p* < 0.001) and marital status (*B*=-0.206, *t*=-2.391, *p* < 0.05) were both negatively linked to IADL. The interaction effect of HCBHS utilization and marital status (*B* = 0.601, *t* = 3.311, *p* < 0.001) was positively correlated with IADL, demonstrating that the relationship between HCBHS utilization and IADL was moderated by marital status. Furthermore, IADL (*B* = 0.566, *t* = 16.058, *p* < 0.001) and HCBHS utilization (*B*=-0.541, *t*=-2.702, *p* < 0.01) were positively and negatively related to depressive symptoms, respectively. Additional details of the other covariates are displayed in Supplementary Table 1.

**Table 3:** Correlation between HCBHS utilization and depressive symptoms with IADL as a mediator and marital status as a moderator

Model pathways	R^2^	B	SE	t	95%CI
HCBHS utilization→IADL	0.187	-0.616	0.162	-3.801	(-0.934, -0.298)
Marital status→IADL		-0.206	0.086	-2.391	(-0.375, -0.037)
HCBHS utilization×Marital status→IADL		0.601	0.182	3.311	(0.245, 0.957)
IADL→Depressive symptoms	0.211	0.566	0.035	16.058	(0.497, 0.635)
HCBHS utilization→Depressive symptoms		-0.541	0.200	-2.702	(-0.934, -0.149)

Note: IADL, instrumental activities of daily living; HCBHS, home and community-based healthcare services; B, regression coefficient; SE, standard error; CI confidence interval

**Fig. 3:**
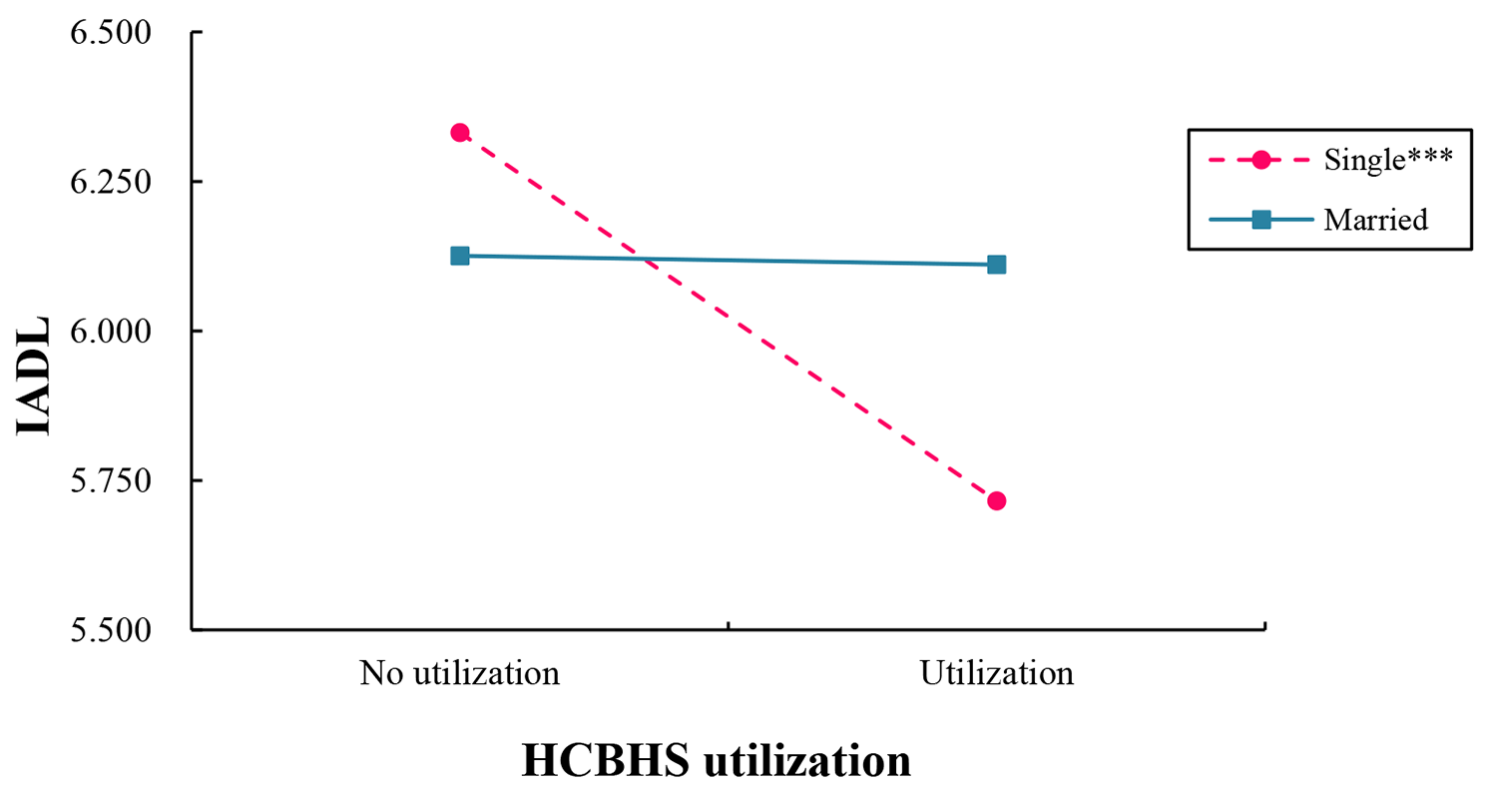
Moderation of marital status on HCBHS utilization and IADL disabilities Note. IADL, instrumental activities of daily living; HCBHS, home and community-based healthcare services; ^***^p < 0.001

The outcome of the simple slope examination is presented in Table 4; Fig. 3, which shows that HCBHS utilization was negatively linked to IADL among single respondents (*B*_simple_=-0.616, *t*=-3.801, *p* < 0.001). However, among married respondents, HCBHS utilization (*B*_simple_=-0.015, *t*=-0.180, *p =* 0.858) was not associated with IADL.

**Table 4:** The impact of HCBHS utilization on IADL based on marital status

Marital status	B	SE	t	p	BootLLCI	BootULCI
Married	-0.015	0.082	-0.180	0.858	-0.176	0.147
Single	-0.616	0.162	-3.801	<0.001	-0.934	-0.298

Note: B, regression coefficient; SE, standard error; LL, low limit; CI, confidence interval; UL, upper limit

## Discussion

This was the first study to demonstrate that the mediating impact of IADL on the association between HCBHS utilization and depressive symptoms in rural community-dwelling older adults in China could be partially moderated by marital status. IADL did not mediate the association between HCBHS and depressive symptoms among married respondents, however, IADL worked as a mediator among single respondents. These findings shed light on the mechanisms underlying the relief of depressive symptoms in HCBHS utilization and furnish a reference point for promoting the mental health of rural older adults with different marital statuses.

Literature from countries other than China has shown that community-based healthcare services are closely correlated with depressive symptoms among older adults in the US and the Netherlands [10, 37–39]. This study, focusing on Chinese rural older adults, concluded accordingly that HCBHS utilization has an increased likelihood of alleviating depressive symptoms. The circumstances that older adults in rural China might not be well cared for and have poor health status indicated that they have a higher need for HCBHS [40–42]. HCBHS can be effective in promoting the physical function and mental states of Chinese older adults in rural areas [43, 44], and then a sense of life satisfaction and safety will be planted at the bottom among their hearts, resulting in fewer depressive symptoms.

**Fig. 4:**
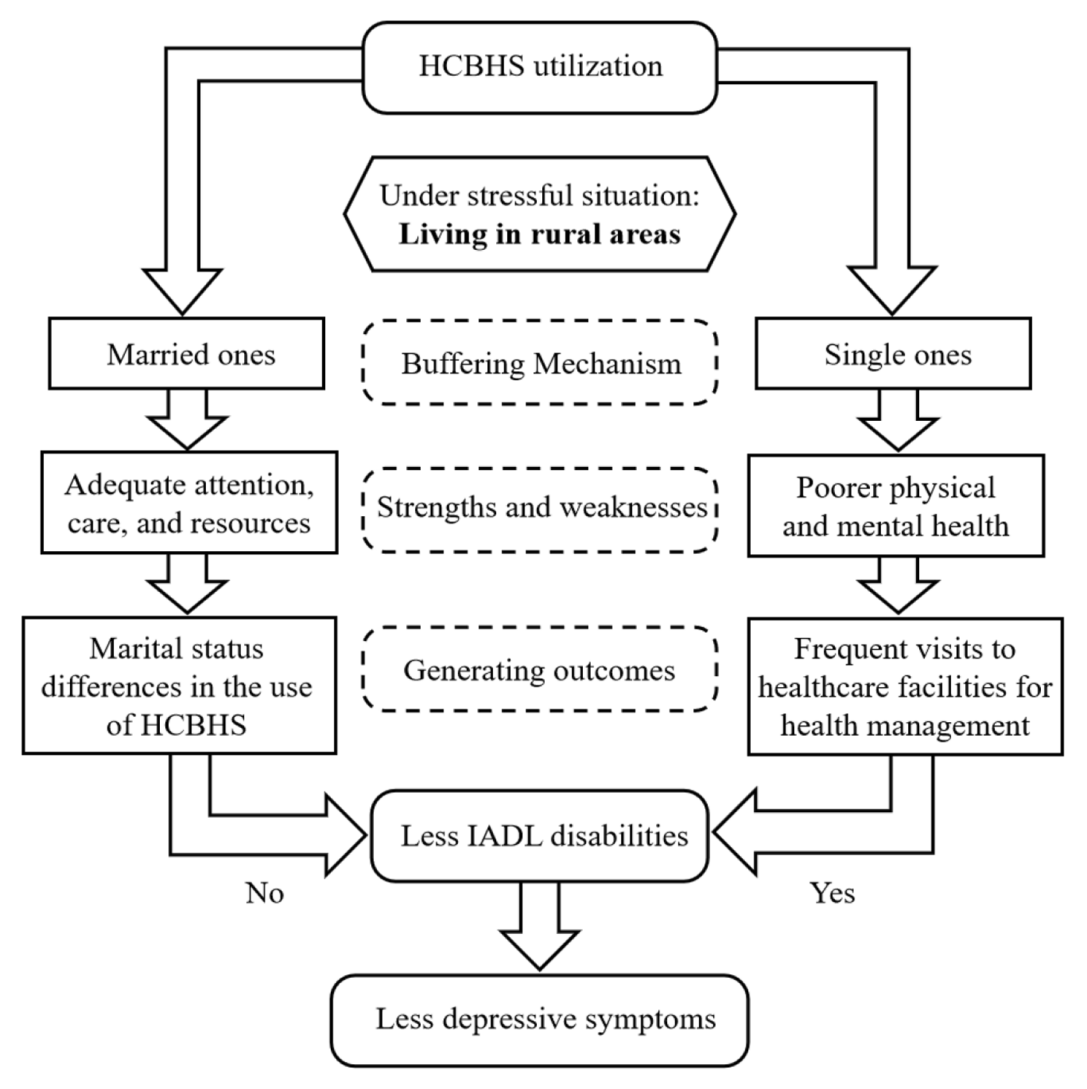
Conceptual framework of marital status differences in HCBHS utilization easing IADL disabilities and depressive symptoms Note. IADL, instrumental activities of daily living; HCBHS, home and community-based healthcare services

Furthermore, we concluded that IADL had a mediating effect on the association between HCBHS utilization and depressive symptoms among single but not married respondents. Spouses, the basis of long-term social ties and networks, may act as a buffering mechanism when older adults live in rural areas [13, 45]. The buffering effect theory suggests that social support benefits individuals’ physical and mental health under stressful circumstances, such as living in rural areas. Figure 4 elaborates on the reasons for the marital status difference in the extent to which HCBHS reduce IADL disabilities among rural community-dwelling older adults in China, from the perspective of spouses as a buffering mechanism.

Not all rural older adults who use HCBHS are likely to reduce their IADL disabilities. Married rural older adults would receive more attention, care, and resources than their single counterparts, and their spouse can supervise and correct inappropriate health behaviors, such as preventing them from alcohol abuse and controlling their daily smoking, which can alleviate IADL disabilities [46]. Given relatively adequate social support from partners, rural married older adults would tend to reduce HCBHS utilization, making it less effective in alleviating IADL disabilities. Therefore, HCBHS utilization was not associated with IADL disabilities among rural older adults within marital union.

The alleviation effect of HCBHS on depressive symptoms in single rural Chinese older adults was affected by IADL limitations. Single rural community-dwelling older adults lacked companionship and were more likely to remain in poorer physical and mental health and devoid of social participation than those with a spouse [47]. In addition, rural community-dwelling older adults without a spouse were more motivated to visit community-based healthcare facilities for health management and comprehensive care, use more HCBHS, and meet their interpersonal interaction needs, thus reducing IADL disabilities and alleviating depressive symptoms [48]. Therefore, HCBHS might not work to reduce IADL disabilities in married community-dwelling older adults living in rural areas compared with those who were single.

In summary, marital status was not only correlated with IADL disabilities but also moderated the mediating influence of IADL, in which HCBHS utilization was associated with IADL disabilities among single but not married respondents.

## Limitations

This study had several limitations. First, the cross-sectional project design of this study could not confirm the causal relationship among the variables. Longitudinal studies should further assess the underlying mechanisms of the association between HCBHS utilization and depressive symptoms. Second, the study only examined the mechanisms underlying the correlation between HCBHS utilization and depressive symptoms in older adults and did not consider other mental health-related factors, such as anxiety and loneliness. Future studies should include a more comprehensive analysis of psychological distress. Moreover, in this study, physical function limitations only considered IADL, not BADL. Future studies should consider both IADL and BADL. Eventually, this study focused on the mechanisms underlying the effects of overall HCBHS utilization on depressive symptoms in older adults in rural China. Future research could further elucidate the mechanisms underlying the effects of specific types of HCBHS utilization on the alleviation of depressive symptoms in older adults.

## Conclusion

Overall, marital status moderated the mediating effect of IADL on the correlation between HCBHS utilization and depressive symptoms in community-dwelling older adults in rural China, with IADL partially mediating this association among single but not among married individuals. Therefore, the government should increase the provision of HCBHS to effectively alleviate depressive symptoms in rural older adults. For single rural older adults with IADL disabilities, the government should provide targeted HCBHS to lessen the level of IADL disabilities and depressive symptoms.

## Electronic supplementary material

Below is the link to the electronic supplementary material.

Supplementary Material 1

Supplementary Material 2

## Data Availability

The data are available at http://charls.pku.edu.cn.

## List of Abbreviations

HCBHS: home and community-based healthcare services
IADL: instrumental activities of daily living
BADL: basic activities of daily living
CHARLS: China Health and Retirement Longitudinal Study
M: mean
SD: standard deviation
SE: standard error
CI: confidence interval
