# Supplementary material for: The relationship between home and community-based healthcare services utilization and depressive symptoms in older adults in rural China: a moderated mediation model
Source: BMC Public Health. 2023 May 30;23:1014. doi: 10.1186/s12889-023-15590-2 (PMC10227957; doi:10.1186/s12889-023-15590-2)
Supplement: Supplementary file 1 — Supplementary Material 1 [file 12889_2023_15590_MOESM1_ESM.pdf]

Supplementary Table 1. Correlations among all variables

Variables	1	2	3	4	5	6	7	8	9	10	11	12	13
1 Gender	1												
2 Age	0.025	1											
3 Education	-0.389***	0.109***	1										
4 Marital status	0.148***	-0.221***	-1.121***	1									
5 Income	0.160***	-0.128***	-0.095***	0.090***	1								
6 Social insurance	0.033*	-0.031*	-0.055***	0.074***	0.008	1							
7 Self-rated health	0.083***	-0.033*	-0.069***	0.051***	0.133***	0.012	1						
8 IADL	-0.099***	0.145***	0.112***	-0.077***	-0.138***	-0.023	-0.336***	1					
9 Smoking	0.492***	-0.025	-0.185***	0.048***	0.121***	-0.004	0.063***	-0.065***	1				
10 Drinking	0.406***	-0.052***	-0.189***	0.088***	0.142***	0.020	0.123***	-0.137***	0.283***	1			
11 Exercising	0.029*	-0.113***	-0.063***	0.052***	0.048***	0.042**	0.087***	-0.245***	0.015	0.087***	1		
12 HCBHS utilization	0.008	0.045**	-0.023	-0.036**	-0.023	0.022	-0.013	-0.024	-0.029*	0.000	0.058***	1	
13 Depressive symptoms	-0.196***	0.005	0.130***	-0.114***	-0.148***	-0.019	-0.372***	0.314***	-0.074***	-0.132***	-0.051***	-0.034**	1

Note. IADL, instrumental activities of daily living; HCBHS, home and community-based healthcare services; *p<0.05, **p<0.01, ***p<0.001.
