# Supplementary material for: The relationship between home and community-based healthcare services utilization and depressive symptoms in older adults in rural China: a moderated mediation model
Source: BMC Public Health. 2023 May 30;23:1014. doi: 10.1186/s12889-023-15590-2 (PMC10227957; doi:10.1186/s12889-023-15590-2)
Supplement: Supplementary file 2 — Supplementary Material 2 [file 12889_2023_15590_MOESM2_ESM.pdf]

Supplementary Table 2. Results of covariates of the moderated mediation analysis.

Control Covariates	B	SE	t	95%CI
Outcome: IADL				
Gender	-0.105	0.074	-1.405	(-0250, 0.041)
Age	0.691***	0.088	7.843	(0.519, 0.864)
Education level	0.225***	0.068	3.327	(0.093, 0.358)
Income	-0.423***	0.083	-5.101	(-0.586, -0.261)
Social insurance	-0.027	0.165	-0.161	(-0.350, 0.297)
Self-rated health	-1.555***	0.062	-24.856	(-1.678, -1.432)
Smoking	-0.001	0.072	-0.003	(-0.142, 0.141)
Drinking	-0.265***	0.068	-3.918	(-0.398, -0.132)
Exercising	-1.534***	0.093	-16.452	(-1.717, -1.352)
Outcome: Depressive symptoms				
Gender	-1.951***	0.201	-9.693	(-2.346, -1.557)
Age	-0.741**	0.236	-3.135	(-1.204, -0.278)
Education level	0.526**	0.185	2.848	(0.164, 0.888)
Income	-1.243***	0.227	-5.488	(-1.687, -0.799)
Social insurance	-0.207	0.449	-0.461	(-1.086, 0.673)
Self-rated health	-4.162***	0.179	-23.231	(-4.513, -3.811)
Smoking	0.644**	0.197	3.277	(0.259, 1.029)
Drinking	-0.162	0.184	-0.877	(-0.523, 0.200)
Exercising	0.677***	0.260	2.604	(0.167, 1.187)

Note: B. regression coefficient; SE, standard error; CI, confidence interval; *p<0.05, **p<0.01, ***p<0.001
